# In Vitro Antiviral Potential of *Syzygium* Species Extracts Against Dengue Virus Serotype 2

**DOI:** 10.3390/biomedicines14061354

**Published:** 2026-06-16

**Authors:** Mohammad Afiq Aizuddin Rosdi, Nur Hana Md Jelas, Bazilah Jusoh, Noorsofiana Padlan, Nurul Hanim Salin, Azimah Amanah, Muhammad Hidhir Khawory, Mohd Ridzuan Mohd Abd Razak

**Affiliations:** 1 Herbal Medicine Research Centre, Institute for Medical Research, National Institutes of Health, Ministry of Health Malaysia, Shah Alam 40170, Selangor, Malaysia; nurhana.mj@moh.gov.my (N.H.M.J.); ridzuan.ar@moh.gov.my (M.R.M.A.R.); 2 Malaysian Institute of Pharmaceuticals and Nutraceuticals, National Institutes of Biotechnology Malaysia, Gelugor 11700, Pulau Pinang, Malaysia; hanim@nibm.my (N.H.S.); azimah@nibm.my (A.A.); muhammad_hidhir@nibm.my (M.H.K.)

**Keywords:** dengue virus serotype 2, *Syzygium myrtifolium*, *Syzygium grande*, high-content screening, antiviral activity

## Abstract

**Background**: Dengue is a vector-borne disease caused by four serotypes of dengue virus (DENV), spread rapidly through Aedes mosquito bites in tropical and subtropical regions. Due to the unavailability of approved antiviral treatment for dengue, continued effort to discover antiviral candidates from natural resources is in need. **Purpose**: In this study, *Syzygium myrtifolium* (stems) and *Syzygium grande* (leaves and stems) extracts were assessed for their cytotoxicity and antiviral activity against DENV2 in vitro. **Methods**: The antiviral properties of methanolic extracts of *S. myrtifolium* (stem) and *S. grande* (leaves and stem) were determined by exposing the serially diluted extracts on DENV2-infected African green monkey kidney (Vero) and human hepatocellular carcinoma (Huh-7). The infection rate was measured by an immunofluorescence-based high-content screening technique. The synthesis of virus progeny was measured by a plaque assay. The ATP-based luminescent assay was used to determine the cytotoxicity effect of the extracts on Vero cells and Huh-7. **Results**: Low cytotoxicity effects of *S. myrtifolium* (stem) and *S. grande* (leaves and stem) extracts were shown in Huh-7 and Vero cells with CC_50_ ranges from >133 to 67.36 μg/mL. The extracts of *S. myrtifolium* stems, *S. grande* leaves and *S. grande* stems showed potent antiviral activities at the pre-infection phase in Vero cells with EC_50_ of 5.57, 5.37 and 10.13 μg/mL, respectively. At the post-infection phase, *S. myrtifolium* stem and *S. grande* leaf and stem extract treatments also resulted in infection-inhibitory effects with EC_50_ of 15.01, 13.45 and 10.33 μg/mL, respectively. However, reduced antiviral activities of the extracts were observed in Huh-7 cells with EC_50_ ranges from 40.23 to 82.83 μg/mL. **Conclusions**: This study provides scientific evidence on *Syzygium myrtifolium* and *Syzygium grande* as promising candidates for further investigation in the development of dengue antivirals.

## 1. Introduction

Dengue is a vector-borne disease caused by the infection of dengue virus (DENV), a member of the Flavivirus genus that belongs to the Flaviviridae family and consists of a single enveloped positive RNA strand with four circulating serotypes (DENV 1, 2, 3 and 4) [1]. The mosquito vector *Aedes aegypti* is the most common vector compared to other species, *Aedes polynesiensis* and *Aedes albopictus* [2].

Dengue fever is established as an endemic disease across more than 100 countries and regions, primarily concentrated within the tropical and subtropical zones of Asia, Africa, the Americas, Australia, and Malaysia [3]. According to Global Dengue Surveillance (2025), there are more than 7.7 million confirmed cases of dengue spanning over 94 countries worldwide [4]. These figures highlight a significant expansion in the disease’s geographical footprint and a substantial increase in the population at risk, necessitating more robust surveillance and intervention strategies.

Infection with DENV can result in a variant of complications and symptoms such as dengue fever, dengue hemorrhagic fever, and dengue shock syndrome [5]. However, as of today, no specific anti-dengue drug is available on the market, and therapies are limited to symptomatic supportive treatment. Although there are three leading dengue vaccines, Dengvaxia, Qdenga and TV003, the need for a dengue test to establish eligibility (i.e., a prevaccination screening) poses a logistical barrier and the vaccine use is only limited to people with a previous DENV infection [6]. Therefore, there is a need to search and develop potent and effective therapeutics to better combat DENV especially from natural resources.

In the discovery and development of antiviral drugs, lead compounds from natural resources such as plants show promising sources that can be used. *Syzygium myrtifolium* Walp is an angiosperm, perennial, evergreen shrub or tree of the Myrtaceae family. Locally, it is known as red wood, red lip, wild cinnamon, ‘kelat oil,’ and ‘kelat paya’ with synonym names such as *Syzygium campanulatum* and *Eugenia oleina* [7]. In addition, *S. myrtifolium* grows from 2 to 20 m and grows widely in India, Myanmar, Thailand, Indonesia, Malaysia, the Philippines, and Singapore. Traditionally, it was found to be used for stomachic purposes [8]. The second plant, *Syzygium grande* (synonym *Eugenia grandis* Wight) is an angiosperm, perennial tree belonging to the family Myrtaceae that grows up to a 50 m height, also known by many vernacular names such as ‘keriang batu’, sea apple, and ‘jambu laut’ [9]. These plants were not extensively studied phytochemically and pharmacologically especially on anti-dengue effects. A previous study on anti-dengue compound candidates revealed that the cyclododecane and n-hexadecanoic acid found in the leaf extracts of *S. grande* were likely the lead compound that imparted the observed inhibitory effect on the DENV2 NS2B-NS3 protease [10]. We hypothesize that the methanolic extracts of *S. myrtifolium* and *S. grande* contain specific bioactive phytochemicals that can inhibit DENV2 infection by either blocking viral entry into host cells or interfering with intracellular viral replication stages, with minimal toxicity to human and primate cell lines. In this study, we performed compound identification by LCMS and phenotypically examined the anti-dengue properties of *Syzygium myrtifolium* and *Syzygium grande* extracts against DENV2 infection in the cell culture system.

## 2. Materials and Methods

**Figure 1:**
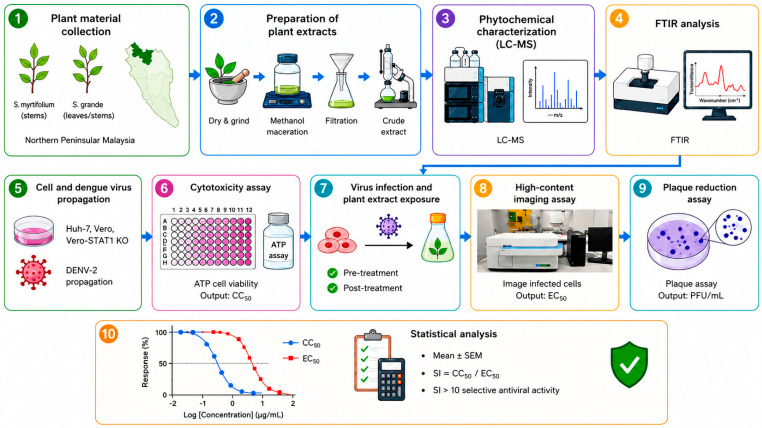
The schematic workflow for the phytochemical characterization and antiviral evaluation of *Syzygium* extracts against DENV2.

The comprehensive experimental workflow, encompassing plant material processing, chemical profiling, and the evaluation of antiviral efficacy against DENV2, is illustrated in Figure 1. Initially, crude extracts were prepared and characterized via LC-MS and FTIR to identify bioactive constituents (Section 2.1, Section 2.2, Section 2.3 and Section 2.4). Subsequently, the safety profile and therapeutic potential of these extracts were evaluated through cytotoxicity and antiviral assays (Section 2.5, Section 2.6, Section 2.7, Section 2.8 and Section 2.9).

### 2.1. Plant Material and Extracts

Plant materials of *Syzygium myrtifolium* (stems) and *Syzygium grande* (leaves and stems) were collected from the Northern Region of Peninsular Malaysia. Mature leaves (fully expanded, dark green, approximately 2–3 months old) were selected to ensure consistency in phytochemical composition. Collection was carried out during the dry season (March–May) to minimize variability due to environmental factors. Identification of the plants was done by Dr. Rahmad Zakaria at the Herbarium Unit, School of Biological Sciences, Universiti Sains Malaysia. Voucher specimens (IPNAT11 and IPNAT45) are deposited at the Herbarium Unit, Natural Product Drug Discovery Centre (NPDC), Malaysian Institute of Pharmaceuticals and Nutraceuticals (IPharm), Penang, Malaysia.

### 2.2. Preparation of Plant Extracts

The plant materials were washed and dried in a drying oven at 40 °C until constant weight was achieved prior to grinding into fine powder. Approximately 100 g of dried plant material was subjected to maceration in 500 mL of methanol (1:5 *w*/*v* ratio) for 72 h at room temperature (25 ± 2 °C). The extraction was performed in sealed glass containers and subjected to continuous agitation using an orbital shaker at 150 rpm to ensure proper solvent penetration and homogenization. After maceration, the mixture was filtered sequentially through a Buchner funnel and Whatman No. 1 filter paper. The filtrate was then concentrated under reduced pressure at 40 °C [11] using a rotary evaporator (EYELA, Tokyo, Japan) to yield a dark-green crude extract. From 100 g of dried plant material, approximately 8 g of crude extract was obtained.

### 2.3. Characterization of Phytocompounds in the Plant Extracts Using Liquid Chromatography-Mass Spectrometry (LC-MS)

A 5000 ppm sample solution was prepared by dissolving 5 mg of the sample in 1 mL of methanol. The solution was subsequently filtered through a 0.22 μm PTFE syringe filter to remove particulate matter prior to LC-MS analysis. Chromatographic separation was performed using an Agilent 1290 Infinity LC system (Agilent Technologies, Santa Clara, CA, USA) equipped with a quaternary pump (G4204A) (Agilent Technologies, Santa Clara, CA, USA), autosampler (HiP Sampler, G4226A) (Agilent Technologies, Santa Clara, CA, USA) and a column compartment (G1316C) (Agilent Technologies, Santa Clara, CA, USA), controlled via MassHunter Workstation Software (Version B.06, Agilent Technologies, Santa Clara, CA, USA). Separation was achieved on a Poroshell 120 EC-C18 column (4.6 × 50 mm, 2.7 μm) (Agilent Technologies, Santa Clara, CA, USA) maintained at 40 °C. The mobile phases consisted of 0.1% formic acid in 10 mM ammonium formate (solvent A) and 0.1% formic acid in acetonitrile (solvent B), delivered at a constant flow rate of 0.2 mL/min. The gradient program was set as follows: 20% B for 0–1 min, increased linearly to 95% B from 1 to 10 min, held at 95% B from 10 to 15 min, decreased to 20% B from 15 to 17 min and equilibrated at 20% B from 17 to 22 min. The injection volume was 2 μL. Mass spectrometric analysis was conducted on an Agilent 6540 UHD Accurate-Mass Q-TOF LC/MS system (Agilent Technologies, Santa Clara, CA, USA) operated in positive electrospray ionization (ESI) mode with a scan range of *m*/*z* 100–1700. The ion source settings were as follows: nebulizer pressure at 35 psi, drying gas flow at 10 L/min, drying gas temperature at 300 °C, sheath gas temperature at 350 °C, and sheath gas flow at 12 L/min. Compounds were detected primarily as protonated ions ([M + H]^+^).

### 2.4. Fourier Transform Infrared (FTIR) Analysis

The plant extracts were evaluated for identifying their chemical bonds or functional groups using FTIR (Perkin Elmer, Waltham, MA, USA) by following the method of Khawory et al. (2023) [12]. Using the pressure arm of an Attenuated Total Reflectance (ATR) crystal, plant extract (2 mg) with 97.5–102.5% (titration) was applied to the crystal’s surface. The Perkin Elmer FT-IR C103470 (Spectrum Two DTGS) (Perkin Elmer, Waltham, MA, USA) was used to analyze the plant extracts in the 4000–450 cm^−1^ (mid-infrared) region at a resolution of 4 cm^−1^ with a scan (16 scans).

### 2.5. Cell and Dengue Virus Propagation

The African green monkey kidney cells (Vero; CCL-81) and Vero-STAT1 knockout cells (CCL-81-VHG) were purchased from the American Type Culture Collection (ATCC, Manassas, VA, USA) and liver cancer cell lines (Huh-7) were purchased from the AddexBio (San Diego, CA, USA). The cells were maintained at 37 °C in a 5% CO_2_ Galaxy 170 S incubator (New Brunswick, Hamburg, Germany) and propagated in Dulbecco’s modified Eagle medium (DMEM 10270; Gibco, Miami, FL, USA) supplemented with 10% fetal bovine serum (FBS) (Gibco, Miami, FL, USA) and 100 U/mL penicillin G and 100 μg/mL streptomycin (Gibco, Miami, FL, USA). A serotype 2 dengue virus isolate was cultured and propagated in Vero-STAT1 cells at 37 °C in DMEM medium with 5% CO_2_. Plaque assay was used to quantify the titer of the dengue virus as mentioned in the study of Mohd Abd Razak et al. [13].

### 2.6. Cytotoxicity Assay

The effect of the plant extracts on cell viability was evaluated by measuring the level of adenosine triphosphate (ATP) using CellTiter-Glo^®^ reagent (Promega, Madison, WI, USA). Briefly, 1 × 10^4^ cell per well of Huh-7 and Vero cells were seeded into a 96-well plate and incubated for 24 h at 37 °C in 5% CO_2_. To prepare the stock solution of extracts, 2 mg of the extracts were weighed and dissolved in 100 µL of DMSO, yielding a final concentration of 20 mg/mL. The various concentrations of extracts were prepared by serial dilution and the final concentration of DMSO in the cells was less than 0.5% (*v*/*v*). After 24 h, the cells were exposed to *S. myrtifolium* and *S. grande* methanolic extracts at various concentrations (0.195–100 µg/mL) for 72 h. Then, CellTiter-Glo^®^ reagent was added to the plate. Luminescence was measured 30 min later using a FLUOstar Omega plate reader (BMG Labtech, Ortenberg, Germany). The percentage of growth inhibition and the half maximal cytotoxic concentration (CC_50_) were determined from a dose–response curve (cell growth versus test items concentrations) generated by using graphpad prism software (Version 10, MA, USA).

### 2.7. Virus Infection and Plant Extract Exposures

The effects of *S. myrtifolium* and *S. grande* methanolic extracts on the DENV2 infection were determined experimentally through the treatment of the extracts at different times. For the pre-treatment assay, 1 × 10^4^ cells/well of Huh-7 and Vero cells were introduced with various concentrations of plant extracts prior to being infected with DENV2 (MOI = 1) for 1 h at 37 °C. Subsequently, the cells were washed with phosphate-buffered saline (PBS) and cultured in DMEM medium containing 2% FBS at 37 °C for 48 h. For the post-treatment assay, Huh-7 and Vero cells were infected with DENV2 (MOI = 1) for 1 h at 37 °C. Then, culture was continued by adding DMEM medium containing 2% FBS with various concentrations of plant extracts or (+)-JNJ-A07 (positive control) (Janssen Pharmaceuticals, Beerse, Belgium). After 48 h, the supernatant of cells was transferred to a new 96 well and stored at −80 °C for the plaque assay and the cells were fixed and processed for high-content imaging analysis.

### 2.8. High-Content Imaging Assay

The treated and untreated DENV2-infected Huh-7 and Vero cells were fixed with ice-cold methanol at −20 °C for 15 min and permeabilized with PBS containing 0.1% Tween-20 (PBS-T). After blocking with PBS-T containing 1% FBS (PBS-FT) at RT for 1 h, the cells were incubated with primary antibody, Dengue Virus Type 1–4 Monoclonal Antibody (D1-11(3)) (1:200) (Invitrogen, Waltham, MA, USA), at 4 °C overnight. After the cells were washed with PBS-T, they were incubated with secondary antibody, Alexa Fluor^TM^ 488 anti-mouse antibody (1:1000), and nucleus-stained with Hoechst solution (1:2500) (Invitrogen, Waltham, MA, USA) at RT for 1 h. Fluorescence was visualized using an Operetta high-content image analysis system (Perkin Elmer, Waltham, MA, USA). The percentage of viral infection and the half maximal effective concentration (EC_50_) were determined from a dose–response curve (infection percentage versus test items concentrations) generated by using graphpad prism software.

### 2.9. Plaque Reduction Assay

A plaque reduction assay was carried out to determine the progeny synthesis of DENV2 after treatment with the extract. Briefly, a 10-fold serial dilution of the culture supernatant (diluted with HBSS solution) was added to a monolayer of Vero cells that had been seeded with 4.5 × 10^5^ cells/well overnight. The plate was incubated at RT for 1 h with swirling every 15 min. Subsequently, the culture supernatant was aspirated and complete agarose media containing 1× DMEM media and 10% FBS was overlayed on the plate. The plate was incubated once the agarose hardened at 37 °C in CO_2_ for 6 days. The Vero cell monolayers were stained with 1% crystal violet in 20% ethanol leaving clear unstained plaques resulting from virus infection.

The infectivity titer was expressed as the number of plaque-forming units per ml (*PFU*/mL).

$$PFU/mL=\frac{average\;of\;plaques\;number}{D\times V}$$

where

*D* = dilution;

*V* = volume of diluted virus added to the plate.

### 2.10. Statistical Analysis

Data are presented as the mean ± SEM of three independent experiments conducted in duplicate. The selectivity index (SI) was calculated as the ratio of CC_50_ (50% cytotoxic concentration) to EC_50_ (50% effective concentration). CC_50_ was determined using an ATP-based assay, while EC_50_ was obtained from antiviral dose–response curve analysis. SI values were calculated using the formula SI = CC_50_/EC_50_. An SI value greater than 10 was considered indicative of selective antiviral activity.

## 3. Results and Discussion

### 3.1. Characterization of Phytocompounds

**Figure 2:**
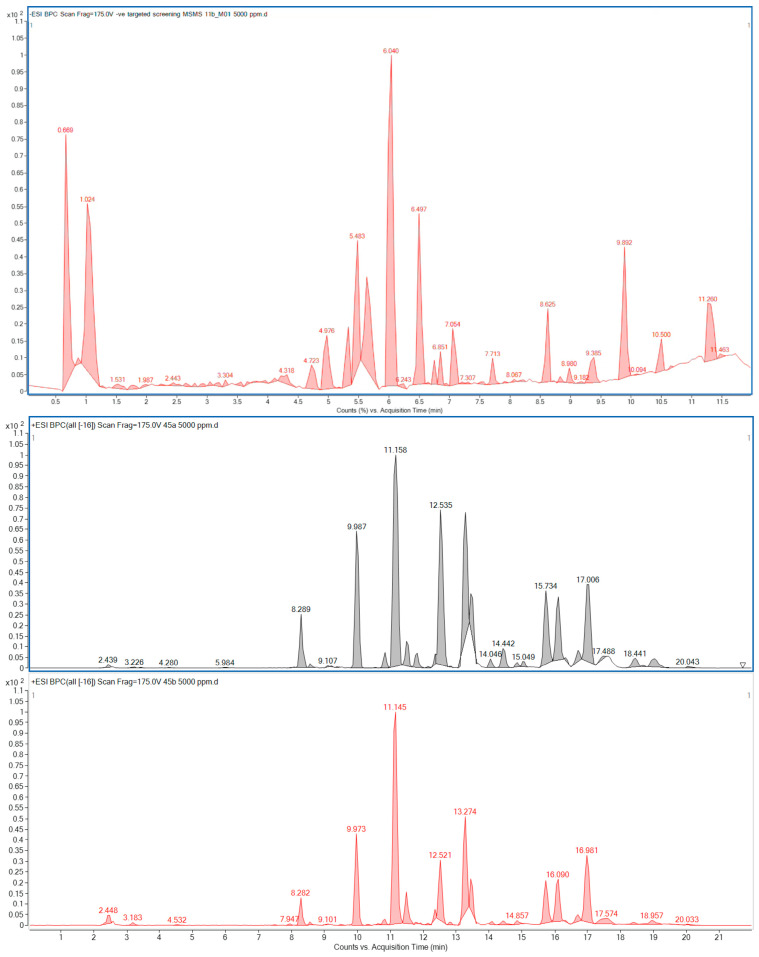
Compound profiling of *S. myrtifolium* stem (**above**) and *S. grande* leaf (**middle**) and stem (**below**) extracts using Q-TOF LC/MS.

The presence of various phytochemicals with anti-dengue properties, such as flavonoids, in numerous plants has been extensively studied [14]. In this study, the major phytocompounds found in the methanolic extract of *S. myrtifolium* stems and *S. grande* leaves and stems as shown in Figure 2 and Table 1. As shown in Table 1, *S. myrtifolium* stem extract contains various bioactive compounds such as quinic acid and ellagic acid, which have previously been shown to exhibit significant anti-DENV2 effects. Quinic acid derivatives demonstrated anti-dengue effects by interrupting the dengue replication stage [15]. Moreover, the ellagic acid in this extract potentially impaired virus attachment to the host cell, as ellagic acid showed a good docking score with all the four glycoproteins from dengue 1–4 viruses [16]. The interaction of compounds with binding sites on the E protein, specifically those involved in cellular membrane interactions, can disrupt the adsorption and fusion processes. This interference prevents the virus from penetrating the cellular cytoplasm and subsequently inhibits its replication [17].

On the other hand, *S. grande* extracts contain various compounds such as myricetin, myricitrin and β-sitosterol, which were also previously isolated by Samy et al. (2014) [18]. The anti-dengue properties of myricetin were found to act as non-competitive inhibitors for the NS2b/NS3 protease enzyme [19,20,21] while β-sitosterol may block the viral entry by inhibiting the fusion process by forming strong interactions with the DENV E glycoprotein [22].

**Table 1:** Phytochemical compounds of *S. myrtifolium* stem and *S. grande* leaf and stem extracts.

No	Compound	Formula	Retention Time, RT	Mass (*m*/*z*)	Previous Studies on Anti-Dengue Compound Candidates
*S. myrtifolium* stem					
1	quinic acid	C_7_H_12_O_6_	0.67	191.056	[15]
2	bergenin	C_14_H_16_O_9_	1.02	327.072	
3	4-O-methyl gallate	C_8_H_8_O_5_	2.04	183.030	
4	rutin	C_27_H_30_O_16_	5.53	609.146	
5	quercetin-3-galacturonide	C_21_H_18_O_13_	5.53	477.068	
6	ellagic acid	C_14_H_6_O_8_	5.64	300.999	[16]
7	isoquercitrin	C_21_H_20_O_12_	5.69	463.089	
8	avicularin	C_20_H_18_O_11_	5.88	433.078	
*S. grande* leaves & stem					
1	myricetin	C_15_H_10_O_8_	4.71	319.0447	[19,20,21]
2	myricitrin	C_21_H_20_O_12_	6.24	465.1024	
3	myricetin 3-O-β-D-glucopyranoside	C_21_H_20_O_13_	6.79	481.0977	
4	quercetin	C_15_H_10_O_7_	8.92	302.0424	
5	alpinetin	C_16_H_14_O_4_	9.97	271.0972	
6	caffeic acid	C_9_H_8_O_4_	11.17	181.0495	
7	β-sitosterol	C_24_H_30_O_6_	12.32	415.2101	[22]
8	2′,6′-dihydroxy-4′-methoxychalcone	C_16_H_14_O_4_	12.44	271.0965	
9	alpinetin methyl ether	C_17_H_19_O_4_	13.19	285.1118	
10	officinoside B	C_19_H_32_O_8_	15.70	389.2113	
11	5-methoxy-7-prenyloxy-8-C-prenylflavanone	C_26_H_30_O_4_	16.02	407.2221	
12	4′-O-Methylbavachalcone	C_22_H_24_O_4_	17.01	353.1750	

**Figure 3:**
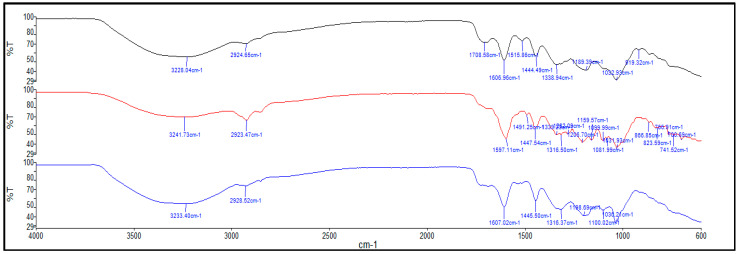
FTIR spectrum of *S. myrtifolium* stem (black), *S. grande* leaf (red) and *S. grande* stem (blue) extracts.

The chemical bonds or functional groups present in the plant extracts were predicted using FTIR. The bonds were determined by interpreting the infrared absorption spectra. Figure 3 shows the FTIR spectrum of the plant extracts, while Table 2 shows the interpretation of the functional groups in the plant extracts.

Strong bonds were found at 1607 cm^−1^ in *S. myrtifolium* stem extract while *S. grande* extracts showed peaks at 919 cm^−1^ (leaves) and 1607 cm^−1^ (stem) were strong. These results demonstrated the presence of phenol, alkane, ester, methylene and methyl groups in these plant extracts.

### 3.2. Cytotoxicity Effect of S. myrtifolium and S. grande Extracts

**Figure 4:**
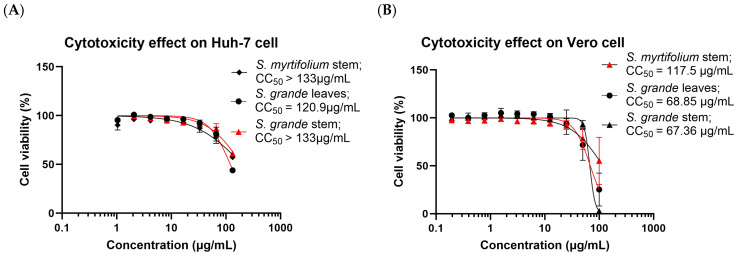
Dose–response curves showing the cytotoxicity effects of *S. myrtifolium* and *S. grande* extracts on the (**A**) Huh-7 and (**B**) Vero cells. The data shown are the mean ± SEM from three independent experiments.

The cytotoxicity assay was evaluated using CellTiter-Glo^®^ reagent which determines the number of viable cells in culture based on quantitation of the ATP present, an indicator of metabolically active cells. The assay was performed on the Huh-7 and Vero cells after 72 h of treatment with the plant extracts. Based on the results in Figure 4, all the extracts showed low cytotoxicity effects on Huh-7 cells with CC_50_ values of more than 120 µg/mL (>120 µg/mL). The results were similar to those reported by Zakaria et al. (2019) [10] which showed these plant leaf extracts have a low cytotoxicity effect on SV-HUC-1 and HTB-4 cells with IC_50_ values of >120 and >250 µg/mL, respectively. These results indicated that the extracts were non-toxic since the value was more than 20 µg/mL [23,24].

Figure 4 shows the results in which no cytotoxicity effects (CC_50_ > 100 µg/mL) were observed after treatment with *S. myrtifolium* on the Vero cells. However, higher cytotoxic effects were observed after treatment with *S. grande* leaf and stem extracts on the Vero cells with CC_50_ of 68.85 µg/mL and 67.36 µg/mL, respectively.

### 3.3. Antiviral Activity of S. myrtifolium and S. grande Extracts Against DENV2 Infection

**Figure 5:**
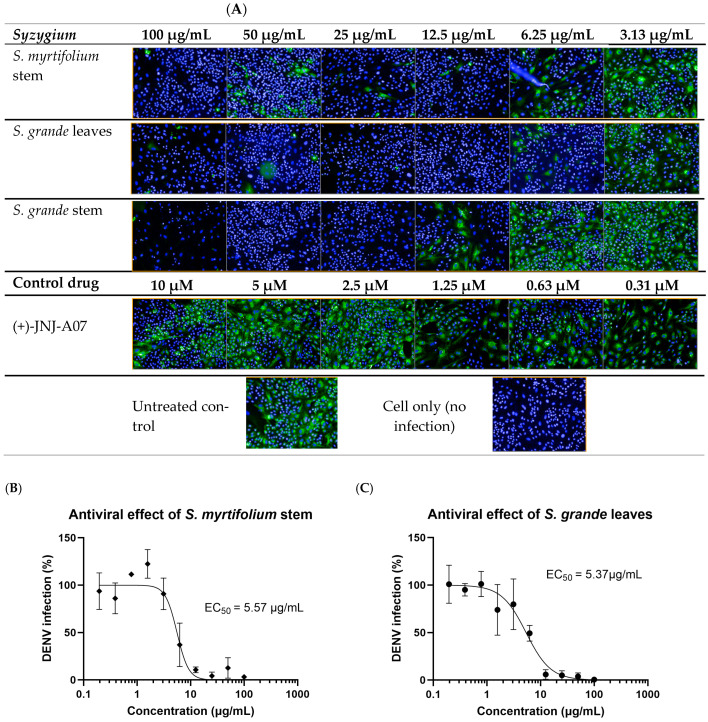

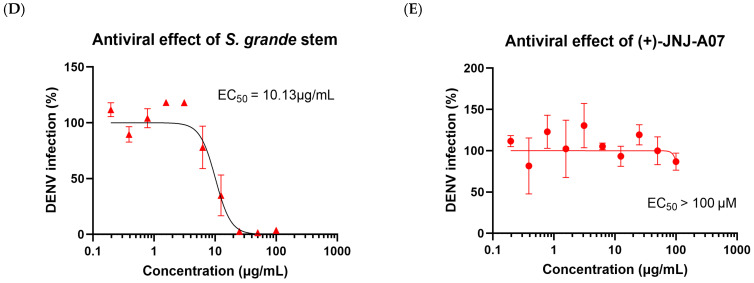
The antiviral effects of *S. myrtifolium* and *S. grande* in the pre-treatment assay against DENV2 infection in the Vero cells. (**A**) High-content screening of the plant extracts compared to the (+)-JNJ-A07 drug and (**B**–**E**) EC_50_ determination of the plant extracts on antiviral activity. The data are expressed as the mean ± SEM of three independent experiments.

**Figure 6:**
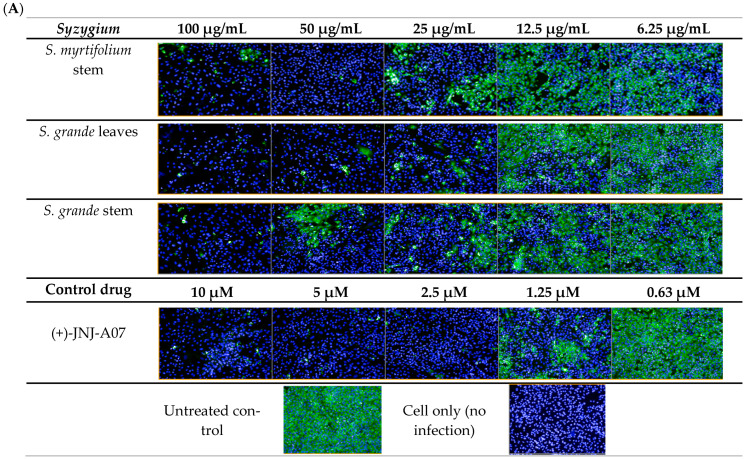

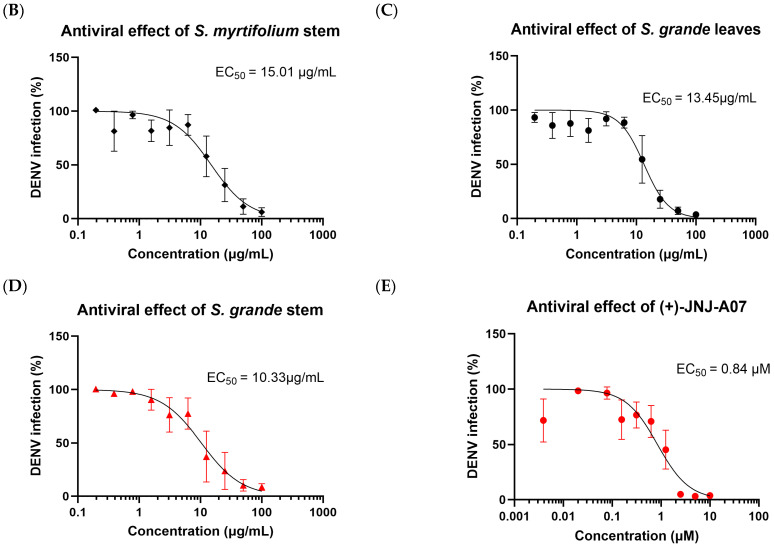
The effects of *S. myrtifolium* and *S. grande* during the post-treatment assay against DENV2 infection in the Vero cells. (**A**) High-content screening of the plant extracts compared to the (+)-JNJ-A07 drug and (**B**–**E**) EC_50_ determination of the plant extracts on antiviral activity. The data are expressed as the mean ± SEM of three independent experiments.

Antiviral studies commonly aim to discover compounds that interfere at different stages in the viral infection process. To explore this, the pre-treatment assay was used to determine the capability of the plant extracts to block the viral adsorption to the cell and thereby protect the organism against DENV infection, while post-treatment assay was used to determine the capability of plant extracts to block the intracellular replication of DENV. The results in Figure 5 show that the pre-treatment of the plant extracts reduced the DENV2 infection, in which the stem extract of *S. myrtifolium* and the leaf extract of *S. grande* showed higher inhibition activity with half maximal inhibition concentration (EC_50_) values of 5.57 and 5.37 µg/mL, respectively, while the *S. grande* stem extract showed an EC_50_ value of 10.13 µg/mL. The positive control drug (+)-JNJ-A07, which is the NS4-NS3B blocker, did not inhibit the virus infection in the pre-treatment assay (Figure 5) because it is mainly active in the intracellular viral replication stage [25]. In the post-treatment assay, the plant extracts also showed a reduction in DENV2 infection albeit at higher EC_50_ values such as 13.45 µg/mL (*S. grande* leaves), 10.33 µg/mL **(***S. grande* stem) and 15.01 µg/mL (*S. myrtifolium* stem). These results were in agreement with the previous study demonstrating the inhibition activity of the extracts on the NS2B-NS3 protease enzyme with exceptional inhibition activity of >95% with corresponding IC_50_ values ranging within 2–5 µg/mL, hence affecting the virus replication process [10]. Indeed, the NS4-NS3B blocker drug (+)-JNJ-A07 showed potent antiviral activity in the post-treatment assay, scoring an EC_50_ value of 0.84 µM (Figure 6).

The higher infection reduction activity in the pre-treatment assay demonstrated that the plant extracts may have the capability to prevent the DENV2 virus from attaching to and entering host cells. However, further assessment needs to be carried out to identify its mechanism. This infection-blocking activity can be carried out through a few mechanisms such as targeting host cellular receptors to inhibit virus entry by interfering with the binding of viral proteins, blocking the attachment of the virus to host cells by targeting its structural proteins involved in entry stage and/or focusing on non-structural proteins that are mainly involved in blocking viral replication [26].

**Figure 7:**
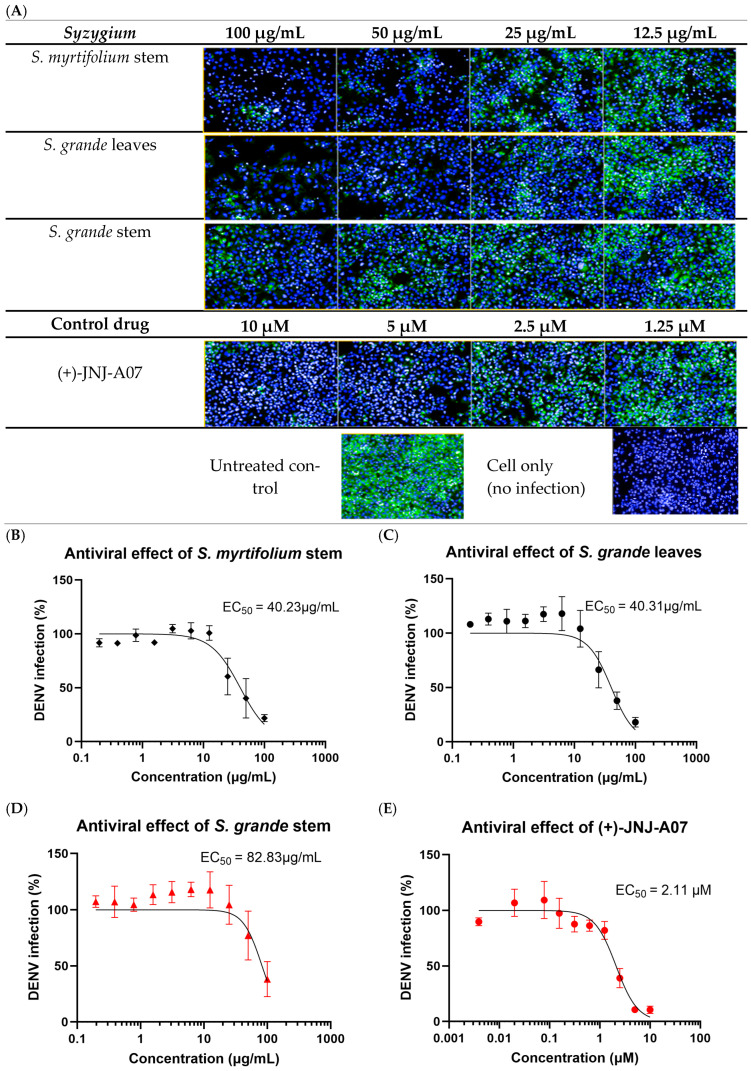
The effects of *S. myrtifolium* and *S. grande* during the post-treatment against the DENV2 infection in the Huh-7 cells. (**A**) High-content screening of the plant extracts compared to the (+)-JNJ-A07 drug and (**B**–**E**) EC_50_ determination of the plant extracts on antiviral activity. The data are expressed as the mean ± SEM of three independent experiments.

Hepatocytes, serving as a primary site for DENV replication and immune modulation, display cytopathic effects (CPEs) such as cellular rounding, detachment, and demise following DENV-2 infections [27]. Thus, the liver cell is crucial as a screening tool for determining drug effectiveness in the drug discovery process. Based on the result in Figure 7, *S. myrtifolium* stem and *S. grande* leaves showed milder antiviral activity with EC_50_ values of 40.23 and 40.31 µg/mL, respectively. Lower antiviral activity was shown by the *S. grande* stem with an EC_50_ value of 82.83. The fact that the Huh-7 cells showed higher EC_50_ values compared to the Vero cell could be due to the biotransformation processes in the liver that reduced the antiviral activity of the extracts. The liver cells are the primary site of drug metabolism in the body which may converts bioactive compounds in extracts into metabolites that are not active for anti-dengue activity. In liver cells, cytochrome P450 enzymes (CYPs) are the primary enzymes responsible for the drug metabolism that affects both drug toxicity and efficacy [28]. Further study needs to be carried out to assess their drug metabolism profile especially through phase I and phase II metabolizing enzymes.

### 3.4. Progeny Synthesis of DENV2

**Figure 8:**
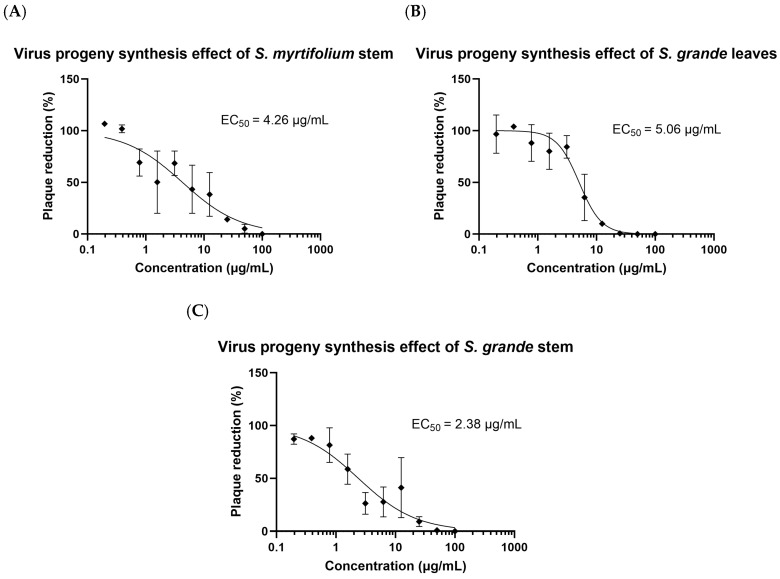
The plaque reduction effects of the *S. myrtifolium* and *S. grande* extracts in the DENV2-infected Huh-7 cells. The data are expressed as the mean ± SEM of three independent experiments (**A**–**C**).

The synthesis of virus progeny was measured by the plaque reduction assay. In this assay, the extracellular virus progeny in the culture supernatants (from the post-treatment assay in the Huh-7 cells) was quantitated. Based on the result (Figure 7), the decreased amounts of viral plaques toward higher concentration indicated for *S. myrtifolium* stem, *S. grande* leaf and *S. grande* stem extracts also affected the viral progeny production with EC_50_ values of 4.26, 5.06 and 2.38 µg/mL, respectively. Although the infection-blocking activity was low (Figure 8), the extracts showed their potential in affecting the production and release of infective viral progeny in the Huh-7 cells.

The selectivity index (SI) was employed to determine the window between antiviral efficacy and cellular toxicity. A higher SI value indicates a superior safety profile and more potent anti-dengue potential, suggesting the extract can inhibit viral replication and infection without compromising host cell viability. In ethnopharmacological research, an SI >10 is typically considered a benchmark for a plant extract to be deemed a ‘good’ lead for further drug development [29]. Based on Table 3, both *S. myrtifolium* and *S. grande* extracts exceeded this threshold, suggesting that their inhibitory effect on DENV2 is not a result of non-specific cellular stress but rather a targeted antiviral mechanism. The high potency of *S. myrtifolium* and *S. grande* may be attributed to the presence of specific bioactive secondary metabolites common in the *Syzygium* genus which have been mentioned earlier to interfere with the DENV2 NS2B-NS3 protease and viral attachment phases.

## 4. Conclusions

In conclusion, this study demonstrates that *S. myrtifolium* (stem) and *S. grande* (leaf and stem) extracts possess significant in vitro antiviral activity against DENV2. The extracts were particularly effective in the pre-treatment phase, suggesting a potential role in blocking viral attachment or entry. Furthermore, the high selectivity index (SI > 50) observed for progeny synthesis in the Huh-7 cells highlights a favorable safety profile and targeted antiviral action. While biotransformation in the liver cells appeared to reduce overall post-treatment potency, the extracts remained highly effective at reducing the production of infectious viral particles. These findings provide scientific validation for *Syzygium* species as promising biopharmaceutical candidates for the development of novel dengue therapeutics. In addition, these findings justify further investigation into the bioassay-guided fractionation of these extracts to isolate the specific phytochemicals responsible for the anti-dengue activity and to elucidate their exact mechanism of action within the DENV replication cycle.

## Figures and Tables

**Table 2:** FTIR spectral peak values and functional groups.

Plant Extract	Peak Values (cm^−1^)	Functional Groups *	Predicted Compound
*S. myrtifolium* stem	3228	O–H stretch, v	Phenol
	2925	H–C–H stretch, w	Alkane
	1709	C=O stretch, w	Ester
	1607	C–C=C stretch, s	Aromatic Ring
	1444	C–H_2_ bend, m	Methylene Group
	1339	C–H_3_ bend, w	Methyl Group
	1189/1033	C–O stretch, m	Ester
	919	C–H bend, s	Hydrocarbon
*S. grande* leaves	3242	O–H stretch, v	Phenol
	2923	H–C–H stretch, m	Alkane
	1597	C–C=C stretch, s	Aromatic Ring
	1448	C–H_2_ bend, m	Methylene Group
	1339	C–H_3_ bend, w	Methyl Group
	1282	C–C stretch, w	Alkane
	1160/1032	C–O stretch, m	Ether
	867	C–H bend, w	Hydrocarbon
*S. grande* stem	3233	O–H stretch, v	Phenol
	2929	H–C–H stretch, w	Alkane
	1607	C–C=C stretch, s	Aromatic Ring
	1446	C–H_2_ bend, m	Methylene Group
	1316	C–H_3_ bend, w	Methyl Group
	1198/1036	C–O stretch, m	Ether

* v = variable; s = strong; m = medium; w = weak.

**Table 3:** Summary of antiviral activities of *S. myrtifolium* and *S. grande* extracts and selectivity index (SI).

		*Syzygium* Extract		
		*S. myrtifolium* Stem	*S. grande* Leaves	*S. grande* Stem
Antiviral/Cytotoxicity Activity on Vero Cell (µg/mL)	CC_50_	117.5	68.85	67.35
	Pre-treatment (EC_50_)	5.57	5.37	10.13
	Hill slope	−4.01	−1.9	−3.44
	SI value	21.10	12.82	6.65
	Post-treatment (EC_50_)	15.01	13.45	10.33
	Hill slope	−1.40	−2.11	−1.31
	SI value	7.83	5.12	6.52
Antiviral/Cytotoxicity Activity on Huh-7 Cell (µg/mL)	CC_50_	>133	120.9	>133
	Post-treatment (EC_50_)	40.23	40.31	82.83
	Hill slope	−1.81	−2.29	−2.93
	SI value	>3.31	3.00	>1.61
	Progeny synthesis (EC_50_)	4.26	5.06	2.38
	Hill slope	−0.87	−2.27	−0.89
	SI value	>31.22	23.89	>55.88

## Data Availability

The datasets generated and analyzed during the current study are available from the corresponding author upon reasonable request.
